# Family Functioning and Multiple Sclerosis: Study Protocol of a Multicentric Italian Project

**DOI:** 10.3389/fpsyg.2021.668010

**Published:** 2021-06-09

**Authors:** Marialaura Di Tella, Virginia Perutelli, Giuseppina Miele, Luigi Lavorgna, Simona Bonavita, Stefania Federica De Mercanti, Lidia Mislin Streito, Marinella Clerico, Lorys Castelli

**Affiliations:** ^1^Department of Psychology, University of Turin, Turin, Italy; ^2^Department of Clinical and Biological Sciences, School of Medicine, University of Turin, Azienda Ospedaliera Universitaria San Luigi Gonzaga, Turin, Italy; ^3^Department of Advanced Medical and Surgical Sciences, University of Campania Luigi Vanvitelli, Naples, Italy

**Keywords:** family functioning, multiple sclerosis, dyadic relationships, parental bonding, psychological distress, alexithymia, perceived social support

## Abstract

Multiple Sclerosis (MS) is a chronic inflammatory and neurodegenerative disease, which not only affects physical functioning, but is also associated with cognitive impairments and great psychological distress. The combination of those symptoms may have negative consequences on the family functioning of patients with MS, with detrimental effects on both marital relationships and parental bonding. Furthermore, the presence of individual characteristics and of an adequate social support may also contribute to the quality and endurance of family relationships. Particularly, high levels of alexithymia, a personality trait that affects the recognition of a person's own emotions, have been associated with reduced interpersonal communication skills and enhanced anxiety/depressive symptoms. Therefore, the main aim of the present study is to provide an in-depth evaluation of family functioning and related factors in patients with MS and their families. In order to reach this goal, the perceived quality of family functioning, dyadic relationships, and parental bonding will be first investigated. Secondly, the possible associations between the quality of family relationships and the presence of alexithymia, psychological distress, and perceived social support will be examined. Patients with MS and their families who will consent to take part in the study will be asked to provide sociodemographic and clinical information, and to complete a series of questionnaires, presented and uploaded on an online dedicated platform. The final sample will be made up of 300 families, consecutively recruited from the Italian medical centers involved in the project. The results of the present study will shed light on the family functioning of patients with MS, through a comprehensive assessment of the main factors that are associated with family dynamics. A holistic evaluation of those aspects can help clinicians and researchers understand family dynamics in MS population better.

## Background

Multiple Sclerosis (MS) is a chronic inflammatory and neurodegenerative disease, which usually appears between the age of 20 and 40. Depending on the location and extent of lesions, patients experience a variety of disease-related stressful conditions, such as motor weakness, sensory deficit, impaired balance, and urinary disturbance.

In addition to physical symptoms, patients with MS might also report cognitive dysfunctions (Calabrese, 2006; Ehrensperger et al., 2008; Kratz et al., 2017) and high levels of psychological distress (Feinstein, 2004; Siegert and Abernethy, 2005). In a recent systematic review, Chalah and Ayache (2017) have highlighted that psychiatric disorders appear to affect up to 95% of MS population at some point during their lifetime. Particularly, the authors have shown that up to 57% of individuals with MS report high anxiety levels (Pham et al., 2018), whereas depression can be found in up to 50% of patients (Corallo et al., 2019).

The absence of definitive cures for MS can also contribute to the burden of disease, leading to unpredictable exacerbations, unremitting progression, and decades of illness especially for patients with an early onset (Uccelli, 2014).

All those aspects together can have a significant impact on families, negatively affecting marital relationships and parental bonding. Partners and children of patients with MS find themselves dealing with a number of significant challenges, which can have negative consequences on their well-being and quality of life (Steck, 2000; Ehrensperger et al., 2008; Kouzoupis et al., 2010; Donisi et al., 2021).

With regard to intimate relationships, facing the challenges of MS can put a strain on the couple. A recent review has pointed out that MS significantly affects the probability of remaining in the same relationship, with ~66% of relationships ending over the course of disease (Uccelli, 2014). The diagnosis of MS brings couples to face significant changes in terms of physical restrictions, dependency, and economic status. Sexual relations are also usually affected, with a study reporting that only one-third of healthy partners were satisfied or very satisfied with their sex lives (O'connor et al., 2008). Risk factors for a couple's breakup appear to be a late diagnosis (>35 years of age) and the male gender of the healthy partner, whereas an early diagnosis (<36 years of age) and having young children seem to be protective factors against separation (Pfleger et al., 2010). The perceived quality of marital relationships has also important consequences on the psychological well-being of the healthy partners. Indeed, the quality of the relationship has been reported to be a strong predictor of partner's depression, even greater than MS patient's physical functioning (McPheters and Sandberg, 2010). Conversely, high levels of perceived social support appear to significantly predict marital relationship satisfaction, with positive repercussions on both partners' well-being (O'connor et al., 2008).

Similar to marital relationship, MS may also have a strong impact on parental bonding. Children who have a parent with MS often show great emotional difficulties, with high levels of anxiety and depressive symptoms (Yahav et al., 2005, 2007; Pakenham and Bursnall, 2006; Bogosian et al., 2010). Particularly, the category that appears to be the most affected by the MS diagnosis of one parent are adolescents (11–18 years old) (Bogosian et al., 2010). Indeed, the results of a longitudinal study of families with one parent with MS showed that higher depression scores of MS-affected parents correlated with increased adolescents' internalizing symptoms (Bogosian et al., 2016). Similarly, two studies of Yahav et al. (2005, 2007) found that having a parent with MS has a negative impact on adolescents' psychological well-being, with high levels of both separation anxiety and depressive symptoms reported by this category.

Depending on the level of disability of the ill parent, children have often to take on additional responsibility in the family, which ranges from helping with household tasks to providing assistance to the ill parent (Turpin et al., 2008; Bjorgvinsdottir and Halldorsdottir, 2014). For some children, these supplementary familiar responsibilities can result in limiting involvement with friends and in reducing time spent playing or doing homework (Turpin et al., 2008). Children often report being isolated from friends, having severe restrictions in life, and experiencing feelings of stress when caregiving becomes too demanding, especially during periods of disease relapse (Bogosian et al., 2011; Bjorgvinsdottir and Halldorsdottir, 2014). Moreover, children frequently experience feelings of increasing family's isolation and complain of limited time to nurture relationships between healthy family members (Bowen et al., 2011). Protective factors against children's emotional distress appear to be both a positive family functioning and an adequate communication of the illness characteristics (Pakenham and Cox, 2012). Particularly, children who had only partial information about their parent's illness were found to present more social and behavioral problems compared to children who received better detailed information (Paliokosta et al., 2009).

Other factors that can influence the quality and endurance of family relationships rely on individual characteristics. A recent review has pointed out that patients with MS show high levels of alexithymia, a personality trait that affects the recognition and regulation of a person's own emotions, with prevalence ranging from 10 to 53% (Chalah and Ayache, 2017). Difficulties in understanding and regulating one's own emotions may have drastic impact on interpersonal communication (Bird and Cook, 2013), with negative consequences on family functioning and social interaction. Alexithymia has also been found to be a risk factor for the development of affective disorders, enhancing the levels of anxiety and depressive symptoms (Conrad et al., 2009; Tesio et al., 2018). Furthermore, previous studies have shown that also perceived social support may play an important role in family functioning, due to its contribution to increased quality of life and decreased psychological distress in caregivers (Wollin et al., 1999; Sherman et al., 2007). Similarly, patients with MS who receive resources provided by interactions with other people have been shown to report fewer mental health symptoms, a better physical recovery, and higher levels of quality of life (Costa et al., 2012). In addition to those factors, also some clinical features, frequently observed among patients with MS, may affect the quality of family relationships. Particularly, apathy, a complex neurobehavioral syndrome, has recently received growing attention in patients with MS, due to its prevalence rates as high as 35% (Chiaravallotti and De Luca, 2003; Raimo et al., 2014). Apathy is a condition particularly debilitating, characterized by a general reduction in goal-directed behaviors, including self-care and social interactions, which might cause increasing burden in family members (Fritz et al., 2018).

Finally, in assessing family functioning and all the above-mentioned factors, it is impossible not to take account of the current situation, which is affecting all people worldwide. Indeed, in the last year, patients with MS have found themselves facing an additional challenge, caused by the newly discovered form of coronavirus known as SARS-CoV-2. The MS International Federation has announced that some MS therapeutic approaches may increase the risk of SARS-CoV-2 infection or can have a negative impact on the SARS-CoV-2 vaccine (MSIF, 2020).

All those aspects together could exacerbate the emotional and physical burden usually associated with MS treatment and symptomatology, so that it is essential to examine both marital and parental relationships, as well as the other factors that may be associated with family functioning (i.e., marital relationships, parental bonding, alexithymia, psychological distress, perceived social support, and apathy, that is all those constructs that we have identified as possible factors that could influence family functioning) in patients with MS and their families.

### Study Aims and Hypotheses

#### Primary Outcome

The primary outcome of the study is to analyze family functioning in patients with MS and their families. In order to reach this aim, two specific goals will be pursued: (1) to evaluate the subjective point of view of each family member (i.e., patients with MS, partners, and adolescent children) on family functioning; and (2) to examine the quality of marital and parental relationships, separately considered. The hypothesis is that the diagnosis of MS may have a different impact on the perceived family functioning from patients and family members depending on individuals' sociodemographic and clinical characteristics. Indeed, the available evidence seems to suggest that specific factors, such as a late diagnosis (>35 years of age) and the male gender of the healthy partner, could predispose to greater dysfunctional family and romantic relationships (e.g., Pfleger et al., 2010). Also, high levels of disability and apathy reported by patients with MS might be associated with reduced family functioning and increased burden in caregivers (e.g., Figved et al., 2007; Bayen et al., 2015). Similarly, children who perceive a negative family functioning, are in the adolescence phase, and have received inadequate information about the parent's illness characteristics might report poor parental bonding (Pakenham and Cox, 2012).

#### Secondary Outcome

**Figure 1:**
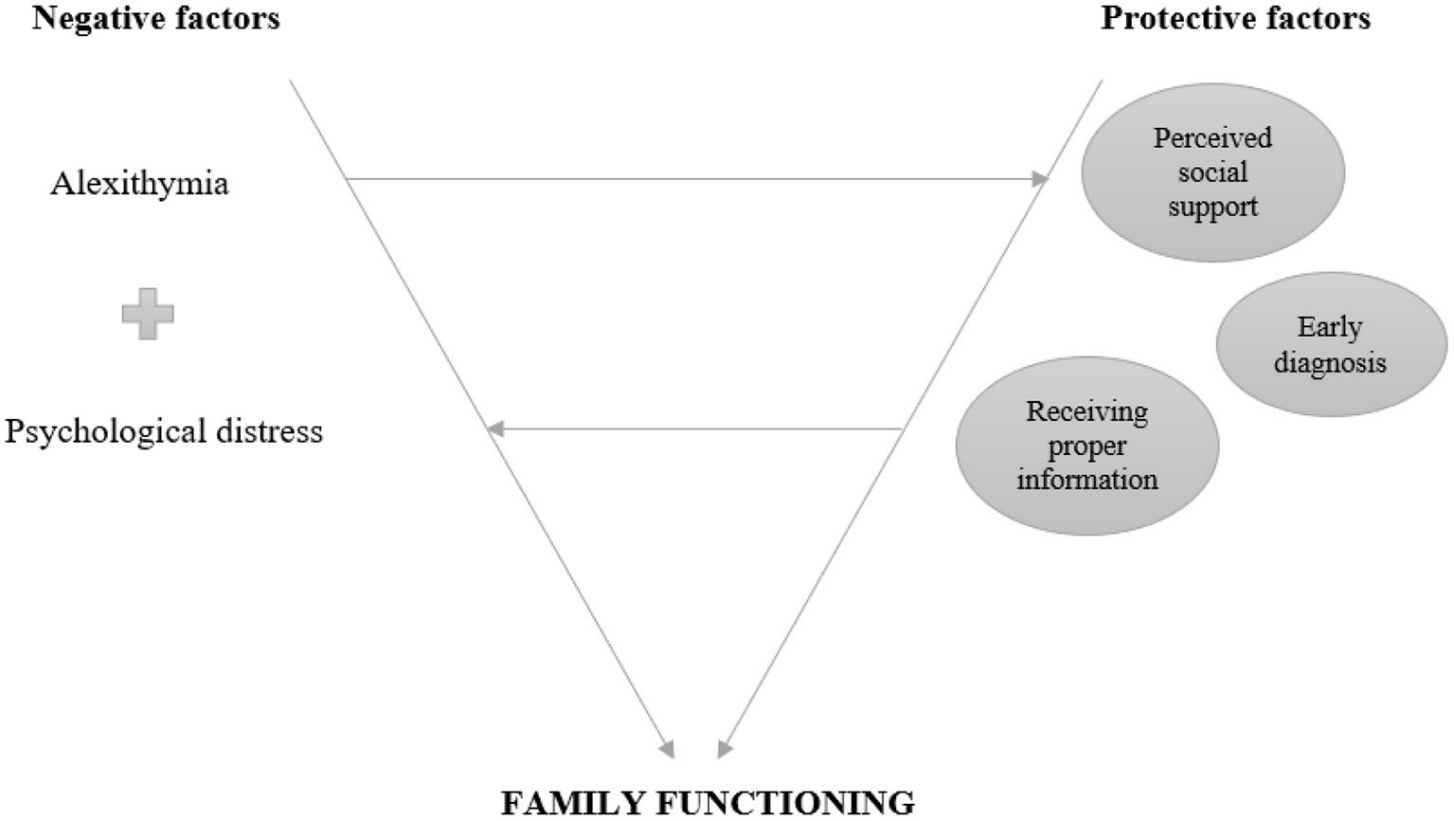
Key factors associated with family functioning in MS.

The secondary outcome of the study is to investigate if the presence of alexithymic traits and psychological distress (anxiety and depressive symptoms), together with the levels of perceived social support, may be associated with the quality of family relationships. The hypothesis is that the presence of alexithymia can influence the way in which individuals react to highly stressful situations, such as the diagnosis of MS. Indeed, greater alexithymic traits have been associated with both higher levels of psychological distress and reduced interpersonal communication skills in clinical and non-clinical populations (Bird and Cook, 2013; Chalah and Ayache, 2017; Di Tella et al., 2018; Saikkonen et al., 2018). Conversely, an adequate perceived social support might function as a protective factor for family ties, helping both the individuals themselves and the family, as a unit, to cope with stressful life experiences, despite the presence of specific individual characteristics (Thoits, 2011). Therefore, we assume that psychological distress could mediate the relationship between alexithymia and family functioning/marital relationship/parental bonding, while perceived social support might moderate the relationship between alexithymia and family functioning/marital relationship/parental bonding (Figure 1).

## Materials and Methods

### Design

The present project is a no-profit, national, multicentric, cross-sectional study involving the following nine sites: Spedali Civili of Brescia—Montichiari Hospital, University “Aldo Moro” (Bari), University of Bologna, University of Cagliari, University of Campania Luigi Vanvitelli (Naples), University “Federico II” (Naples), University of Genoa, University of Turin, Sapienza University of Rome, Tor Vergata University University of Rome. The Department of Clinical and Biological Sciences together with the Department of Psychology of the University of Turin are the coordinating centers, which have originally conceived the study idea and design.

### Recruitment Procedure

The final sample will be made up of 300 families of patients with MS (i.e., 300 patients with MS, 300 partners, and at least 300 adolescent children), consecutively recruited from the Italian medical centers involved in the project. The sample size has been determined based on an a priori power analysis, using the software G^*^Power 3.1 (Faul et al., 2009), with a medium effect size, power of over 0.95, and an alpha level of 0.05, as being sufficient for both repeated measures Analysis of Variance (ANOVA) and *t*-test.

For the present study will be considered both single parent families and nuclear families (consisting of two parents and their adolescent children). Also, subsamples of patients with both relapsing and progressive types of MS will be recruited, in order to consider the different effect that the types of MS could have on the quality of family relationships.

Exclusion criteria will be specifically defined for each family member. With regard to patients with MS, they will be as follows: <18 years old; low educational level (<5 years); insufficient knowledge of the Italian language that would prevent filling in the questionnaires; presence or history of a neurological disorder different from MS or a psychiatric disorder; severe motor or visual impairment that may interfere with psychometric testing; not having children. Exclusion criteria for the partners of people with MS will include: <8 years old; low educational level (<5 years); insufficient knowledge of the Italian language; presence or history of a neurological or psychiatric disorder. For the teenage children, the following exclusion criteria will be used: <12 years old and >18 years; low educational level (<5 years); insufficient knowledge of the Italian language; presence or history of a neurological or severe psychiatric disorder.

All the family members, who will reflect the inclusion criteria (i.e., patients with MS, partners, and adolescent children) and will show interest in taking part in the study, will be given the possibility to complete a battery of questionnaires presented and uploaded on an online platform. For the present study a dedicated platform, managed and supervised by Logic Solution S.r.l., will be employed with the aim of extending the project to as many participants as possible and complying with the GDPR 679/2016.

The dedicated platform will be linked to a completely independent server for filling in the questionnaires (https://www.family.socialnetwork.com/questionari/index/familysn). Users will not be required to enter any personal data and the platform will have no interaction with the original website except through a link.

Doctors of each center will communicate to Logic Solution S.r.l. their mobile numbers, and only they will be authorized to generate access codes for their patients.

A special mobile number will be activated, so that doctors will be able to send, from their number, a text message containing only the mobile number of the patients that would have accepted to participate in the study. If names, surnames or any other words are added, the system will discard the request. Upon receipt of the SMS, the platform will allow: (i) to check if it comes from one of the enabled numbers; in practice, the sender number works like a password, avoiding mailings from unauthorized users or other malicious systems; (ii) to check if the content is only a mobile number; (iii) to generate the access code and send it via SMS to the patient with the same procedure approved. From the platform, it will be possible to download the report of the questionnaires where the number of the doctor will appear in addition to the patient number in such a way as to allow doctors to independently trace the respective users of which Logic Solution S.r.L. will continue not to be aware.

The system is always active and everything happens in a few moments, in order to allow the doctor to enable the patient while he/she is with him/her. Therefore, Logic Solution S.r.l. will be provided only with patients' telephone numbers, in order to be able to send them a text message with a unique alphanumeric code to be used for anonymous access to the questionnaires. This code will be valid not only for the patients themselves, but also for the family members who will agree to participate in the study. Only through that code, which identifies the user in a “strong” way, it will be possible to access the online survey for a limited period of 1 month. Partial saving will be not foreseen, in order not to allow those who might come into possession of the code to see the answers. Therefore, the system will only allow the data to be recorded once all the questions will be answered. The only data recorded will be the answers and the date and the time of access to the questionnaires.

Before completing the questionnaires, the participants will be informed about the nature of the study through informed consent. After giving their informed consent, sociodemographic (i.e., age, gender, educational level, nationality, occupation, marital status, having/not having children, and role in the family unit—patient with MS, partner, adolescent child) and clinical (i.e., disease duration, type of MS, current medication, and Expanded Disability Status Scale—EDSS—for patients with MS only; presence or history of a neurological or severe psychiatric disorder—for all participants) information will be preliminary collected. Participants will then be provided with a different presentation of the questionnaires described below, depending on the answers they will give to the initial questions they will be asked to complete. Indeed, the online platform will allow a ramification of the survey based on the preliminary responses each individual will give (e.g., the role he/she plays in the family unit). Therefore, if the participant identifies himself/herself as a patient with MS, he/she will be directed to the specific questions (i.e., disease duration, type of MS, current medication, and EDSS) and instruments (see below) for this subgroup. Common measures to all family members will include: (1) the short form of the Family Assessment Measure Third Edition; (2) the Hospital Anxiety and Depression Scale; (3) the Multidimensional Scale of Perceived Social Support; and (4) the Toronto Alexithymia Scale. Conversely, specific questionnaires, that is the Dyadic Adjustment Scale and the Inventory of Parent and Peer Attachment, will be administered to patients with MS and their partners (if in a relationship), and to their teenage children (if any), respectively.

### Assessment Instruments

The questionnaires that will be administered for the present study have been previously validated in their online version in a heterogeneous sample of Italian healthy individuals (Lavorgna et al., 2020). The results of this study have indicated that the online versions of the questionnaires we will employ can be considered reliable tools for the assessment of family functioning and related factors.

#### Primary Outcome

In order to reach the primary outcome, family functioning will be assessed with one of the most frequently used self-report instruments, that is the Short form of the Family Assessment Measure, Third Edition (Brief FAM-III, Skinner et al., 2000; Rodrigues and Patterson, 2007; Pellerone et al., 2017). It consists of three modules: the “General Scale,” which evaluates the family as a system; the “Dyadic Relationships Scale,” which examines how each family member perceives his/her relationship with another member; and the “Self-Rating Scale,” which allows each person to rate his or her own functioning within the family. Each scale consists of 14 items scored on a 4-point Likert-type scale, with raw scores ranging from 0 to 15 for the General Scale and from 0 to 18 for the Dyadic Relationship Scale and Self-Rating Scale. For the purposes of the present study, only the Self-Rating Scale will be administered. It has shown good internal consistency with Cronbach's alpha values ranging from 0.80 to 0.88 (Skinner et al., 2000; Pellerone et al., 2017).

The second scale that will be administered to achieve the primary outcome is the Dyadic Adjustment Scale (DAS, Spanier, 1976; Gentili et al., 2002; King and Arnett, 2005), which allows the assessment of dyadic relationship. It consists of four subscales: “Dyadic Satisfaction,” “Dyadic Cohesion,” “Dyadic Consensus,” and “Affectional Expression.” The 32 items of the DAS are answered on a 6-point Likert-type scale, and summing each item across the four subscales it is possible to obtain a total score ranging from 0 to 151. The DAS has shown good internal consistency with Cronbach's alpha scores ranging from 0.70 to 0.95 and test-retest reliability (Carey et al., 1993). Only the DAS total score will be considered for the main data analyses.

The third scale that will be employed to complete the primary outcome is the Inventory of Parent and Peer Attachment (IPPA). The IPPA is a self-report questionnaire developed to assess adolescents' perceptions of the positive and negative affective/cognitive dimension of relationships with their parents and peers (Greenberg et al., 1984). For the present study, the revised version of this scale will be used, which is composed of 75 items, equally divided in three forms: maternal, paternal, and peer (Armsden and Greenberg, 1987; Sieh et al., 2012). Each item is scored on a 5-point Likert-type scale and the total score ranges from 25 to 125 for each subscale. The IPPA has shown good internal consistency (Cronbach's alpha scores: 0.87 for the maternal form, 0.89 for the paternal form, and 0.92, for the peer form) and test-retest reliability. Only the maternal and paternal forms of the IPPA will be considered for the main data analyses.

Finally, for the assessment of apathy in patients with MS only, the Dimensional Apathy Scale (DAS—Apathy) will be administered. The DAS (apathy) is a self-report instrument, which has been recently validated among patients with MS (Raimo et al., 2020). It consists of 24 items scored on 4-point Likert-type scale. The total score ranges from 0 to 72, with higher scores indicating more severe apathy. Three subscale scores can also be derived, each one evaluating different aspects of apathy: “Executive subscale,” which assesses planning, attention, and organization abilities; “Emotional subscale,” which examines emotional integration; and “Behavioral/Cognitive Initiation subscale,” which assesses self-behavior or cognition. The DAS has shown good internal consistency (Cronbach's alpha score: 0.84) and test-retest reliability (Santangelo et al., 2017). Only the DAS (apathy) total score will be considered for the main data analyses.

#### Secondary Outcome

Data on secondary outcome will be collected from three self-report questionnaires.

Particularly, the Hospital Anxiety and Depression Scale (HADS) will be employed to assess symptoms of anxiety and depression (Zigmond and Snaith, 1983; Costantini et al., 1999; Castelli et al., 2009; Honarmand and Feinstein, 2009). It consists of 14 items, seven items for the anxiety subscale (HADS Anxiety) and seven for the depression subscale (HADS Depression), scored in four alternatives ranging from 0 to 3. Two subscale scores (HADS-Anxiety, HADS-Depression) and a total score, ranging from 0 to 21, can be obtained by summing the items. According to Zigmond and Snaith (1983), recommended cut-off scores for each subscale are ≥8, which suggest a clinically significant level of anxiety or depressive symptoms. The HADS has shown good concurrent validity, test-retest reliability and internal consistency (Cronbach's alpha scores: 0.82–0.90) (Bjelland et al., 2002).

The other questionnaire that will be administered for the secondary outcome is the Multidimensional Scale of Perceived Social Support (MSPSS) (Zimet et al., 1988; Prezza and Principato, 2002; Osborne et al., 2007). This scale has been developed to assess perceived social support. The MSPSS consists of 12 items scored on a 7-point Likert-type scale. The total score ranges from 12 to 84 and can be derived by summing the following three subscale scores: “Significant Other,” “Family,” and “Friends.” Higher scores are associated with higher levels of perceived social support. The MSPSS has shown good internal consistency (Cronbach's alpha scores: 0.87–0.94) and test-retest reliability (Osman et al., 2014). Only the MSPSS total score will be considered for the main data analyses.

The last questionnaire that will be employed for the secondary outcome is the Toronto Alexithymia Scale (TAS-20), a self-report instrument designed to assess alexithymia (Taylor et al., 1985; Bressi et al., 1996; Cecchetto et al., 2014). It is made up of 20 items scored on a 5-point Likert-type scale and the sum of all items gives a total score, ranging from 20 to 100, and three subscale scores. Each subscale investigates different aspects of alexithymia: “Difficulty identifying feelings,” “Difficulty describing feelings,” and “Externally-oriented thinking” (Taylor et al., 2003). The scale has shown good internal consistency (Cronbach's alpha coefficients: 0.70) and test-retest reliability (Taylor et al., 2003). Only the TAS total score will be considered for the main data analyses.

### Statistical Analysis

Statistical analyses will be carried out following a multistep method. Before performing any statistical analyses, all variables will be investigated for normality of distribution by visual inspection of plots and indices of asymmetry and kurtosis. Values for asymmetry and kurtosis between −1 and +1 will be considered acceptable in order to prove normal univariate distribution. Measurement invariance (configural, weak, strong, and strict) between the groups (patients with MS, partners, and adolescent children) will also be verified, in order to ascertain the absence of measurement bias.

Descriptive data for patients with MS, partners, and adolescents will be firstly computed, in order to provide an overview of sociodemographic and clinical characteristics of the participants. Descriptive data will be presented as means with standard deviations, for continuous variables, or frequencies with percentages, for categorical variables.

As far as the primary outcome is concerned, repeated measures ANOVA for normally distributed continuous variables, or Friedman test for variables that violate the assumption of normality, will be used to examine mother-father-adolescent differences in perceived family functioning. Paired *t*-test or Mann-Whitney *U* test will be performed, as appropriate, to assess dyadic (i.e., parent-adolescent and couple) differences in perceived parental bonding and marital relationship. Also, one-way ANOVA for normally distributed continuous variables, or Kruskal–Wallis test for variables that violate this assumption will be run to analyze the presence of possible statistically significant differences on FAM-III Self-Rating Scale, DAS total, and IPPA maternal/paternal subscale scores between the subgroups of patients with different types of MS. Finally, one-way or repeated measures Analyses of Covariance (ANCOVAs) will be performed to determine whether group differences on family functioning, marital relationship, and parental bonding would still be significant after controlling for the effect of age, gender, disease duration, EDSS, and DAS (apathy). The effect size will be determined by calculating partial eta-squared (η^2^), Kendall's *W*, Cohen's *d* or Pearson's correlation coefficient *r*, as appropriate. Data will be presented as means with standard deviations or medians with interquartile ranges depending on the distribution of the variables.

With regard to secondary aim, a Structural Equation Model (SEM) will be developed, in order to evaluate the possible role as mediators and moderators of psychological distress (HADS Anxiety and Depression subscale scores) and perceived social support (MSPSS total score) in the relationship between alexithymia (TAS-20 total score) and family functioning (FAM-III Self-Rating Scale score), marital relationship (DAS total score), and parental bonding (IPPA maternal and paternal subscale scores). Particularly, we hypothesize that the relationship between alexithymia and family functioning/marital relationship/parental bonding would be fully or partially mediated by psychological distress. Conversely, we assume that perceived social support would act as a moderator of the relationship between alexithymia and family functioning/marital relationship/parental bonding.

The level of significance for all statistical tests will be set at *p* <0.05. However, the application of specific Bonferroni's corrections for multiple comparisons will be considered, in order to reduce the possibility of committing Type I error. All analyses will be blinded and performed using the Statistical Package for Social Science, version 26.0 (IBM SPSS Statistics for Windows, Armonk, NY, USA: IBM Corp.), and Mplus, version 8.4.

### Ethics and Dissemination

The study involving human participants has been reviewed and approved by AOU San Luigi Gonzaga Ethics Committee (CE 81/2019/U, 24 July, 2019; protocol number 10899; 2 August, 2019). All the participants will provide written informed consent to participate in the study. For participants under the age of 18, written informed consent to participate in this study will be provided by the participants' legal guardian/next of kin.

## Discussion

The present protocol describes the design of a research project aiming to provide an in-depth investigation of family functioning and related aspects in patients with MS and their families (partners and adolescent children).

Previous studies have shown that the diagnosis of MS could have a detrimental effect on family functioning, with negative consequences for marital relationships and parental bonding. Specific factors, such as a late diagnosis, the male gender of the healthy partner and having children in the age of adolescence, appear to be predisposing factors for the development of dysfunctional family relationships (e.g., Uccelli, 2014).

Based on this evidence, our first goal will be to investigate the quality of perceived family functioning, marital relationships, and parental bonding and to examine if the above-mentioned individual sociodemographic and clinical characteristics could be differently related to great vs. poor quality of family relationships.

As a secondary aim, we will assess individual and social factors, such as the presence of alexithymic traits, anxiety/depressive symptoms, and perceived social support, which could play a key role in the way family members relate to each other. Indeed, the available evidence shows that difficulties in understanding one's own emotions seem to be associated with reduced interpersonal communication skills and great psychological distress (Bird and Cook, 2013; Chalah and Ayache, 2017). Conversely, an adequate perceived social support appears to be a protective factor for family ties, with social network providing family members with help to deal with extremely stressful situations, such as a serious chronic medical condition or disability of one of the members (Pakenham and Bursnall, 2006; Jiang et al., 2015). Therefore, while alexithymia and psychological distress can negatively affect family functioning, high levels of social support may play a buffering role in the development of dysfunctional family relationships.

The assessment of all those family functioning-related aspects will be carried out through an *ad hoc* developed web-based platform, which in our opinion represents the most important strength of the present study. By using online methods, data can be easily collected and a high number of participants will thus be enrolled in the study. A second relevant strength of our study is that all the questionnaires we will employ are reliable assessment instruments, which have also been validated for their online administration in a recent published article (Lavorgna et al., 2020). Furthermore, the present project represents the first national multicentric, no-profit study that will be carried out in Italy with a rigorous methodology to evaluate family functioning and related factors in patients with MS.

The present study also presents some limitations that should be acknowledged. Particularly, the cross-sectional design of the study does not allow certain conclusions about causal direction to be drawn. In order to overcome this limitation, future longitudinal studies will be carried out to monitor the impact that the diagnosis of MS can have on family relationships over time. Secondly, only self-report questionnaires will be employed for the present study and this might lead to the underestimation of, for example, the presence of alexithymic traits in individuals falling into borderline cut-off scores. Although performance-based instruments or structured interviews, less dependent on the individuals' awareness, should be preferred to the exclusive use of self-report measures, broad consensual agreement exists between TAS-20 score and observer ratings of alexithymia (Taylor et al., 2000) and evidence shows that subjective and objective measures of emotional awareness are reliably correlated (e.g., Gaigg et al., 2018). Finally, we will not employ distinct instruments for the assessment of anxiety and depressive symptoms (e.g., Raimo et al., 2015), in order to reduce the survey duration. However, we will administer a measure (i.e., the HADS), which has been largely used in different clinical populations and has been previously validated among patients with MS (Honarmand and Feinstein, 2009).

Despite those possible limitations, the present study represents, to the best of our knowledge, the first attempt to provide a comprehensive assessment of family functioning and related factors in patients with MS.

The assessment of all these aspects has become even more central and complex as result of the SARS-CoV-2 infection pandemic. Indeed, various studies have shown that quarantine may have a huge impact on the individuals' psychological well-being and can cause considerable psychological strain (Brooks et al., 2020; Castelli et al., 2020; Holmes et al., 2020). For instance, a recent review has shown that all quarantined individuals reported a high prevalence of psychological distress, with symptoms of anxiety, depression, and post-traumatic stress disorder (Brooks et al., 2020). Similar results have been obtained by assessing mental health symptoms in patients with MS. In fact, in a recent study, patients with MS have been shown to report higher levels of depressive symptoms than healthy controls, suggesting a greater susceptibility of the former to stressors compared to the general population (Costabile et al., 2020).

The available results highlight the inevitable consequences that the pandemic we are currently experiencing has on the mental health of every individual. In patients with MS, whose vulnerability is enhanced both by their chronic condition and by the high prevalence of psychiatric comorbidity they usually present (Feinstein et al., 2014), those negative consequences can even be more significant. Furthermore, patients with MS have found themselves experiencing important disruption to their management of the disease, with many medical appointments being canceled and difficulties in continuing psychotherapy (Costabile et al., 2020).

Taking all these elements into account, it becomes even more important to investigate family functioning and the factors that may be associated with it in MS. A holistic evaluation of the all those aspects can help clinicians and researchers understand family dynamics in MS population better. In this way, it would be possible to plan a better medical management with psychological treatments tailored to the needs of each patient and his/her family.

## Ethics Statement

The studies involving human participants were reviewed and approved by AOU San Luigi Gonzaga Ethics Committee (CE 81/2019/U, 24 July, 2019; protocol number 10899, 2 August, 2019). Written informed consent to participate in this study will be provided by the participants' legal guardian/next of kin.

## Author Contributions

LC, MC, and LL conceived and designed the protocol. MDT, VP, and GM wrote the paper. SB, SFDM, and LMS revised the draft of the paper. All authors contributed to the article and approved the final version of the manuscript.

## Conflict of Interest

The authors declare that the research was conducted in the absence of any commercial or financial relationships that could be construed as a potential conflict of interest. The reviewer SR declared a shared affiliation with several of the authors, GM, LL, and SB, to the handling editor at time of review.
